# A marker of glutathione S-transferase-mediated resistance to insecticides is associated with higher *Plasmodium* infection in the African malaria vector *Anopheles funestus*

**DOI:** 10.1038/s41598-019-42015-1

**Published:** 2019-04-08

**Authors:** Magellan Tchouakui, Mu-Chun Chiang, Cyrille Ndo, Carine K. Kuicheu, Nathalie Amvongo-Adjia, Murielle J. Wondji, Micareme Tchoupo, Michael O. Kusimo, Jacob M. Riveron, Charles S. Wondji

**Affiliations:** 1 Research Unit LSTM/OCEAC, P.O. BOX 288, Yaoundé, Cameroon; 2 Centre for Research in Infectious Diseases (CRID), P.O. BOX 13591, Yaoundé, Cameroon; 3 Department of Animal Biology and Physiology, Faculty of Science, University of Yaoundé 1, P.O. Box 812, Yaoundé, Cameroon; 4 Department of Vector Biology, Liverpool School of Tropical Medicine, Pembroke Place, L35QA Liverpool, UK; 5 University of Douala, P.O. Box 2701, Douala, Cameroon; 6 Centre for Medical Research, Institute of Medical Research and Medicinal Plants Studies (IMPM), P.O. Box 13033, Yaoundé, Cameroon

## Abstract

Metabolic resistance to insecticides is threatening malaria control in Africa. However, the extent to which it impacts malaria transmission remains unclear. Here, we investigated the association between a marker of glutathione S-transferase mediated metabolic resistance and *Plasmodium* infection in field population of *Anopheles funestus* s.s. in comparison to the A296S-RDL target site mutation. The 119F-GSTe2 resistant allele was present in southern (Obout) (56%) and central (Mibellon) (25%) regions of Cameroon whereas the 296S-RDL resistant allele was detected at 98.5% and 15% respectively. The whole mosquito *Plasmodium* and sporozoite infection rates were 57% and 14.8% respectively in Obout (n = 508) and 19.7% and 5% in Mibellon (n = 360). No association was found between L119F-GSTe2 genotypes and whole mosquito infection status. However, when analyzing oocyst and sporozoite infection rates separately, the resistant homozygote 119F/F genotype was significantly more associated with *Plasmodium* infection in Obout than both heterozygote (OR = 2.5; P = 0.012) and homozygote susceptible (L/L119) genotypes (OR = 2.10; P = 0.013). In contrast, homozygote RDL susceptible mosquitoes (A/A296) were associated more frequently with *Plasmodium* infection than other genotypes (OR = 4; P = 0.03). No additive interaction was found between L119F and A296S. Sequencing of the *GSTe2* gene showed no association between the polymorphism of this gene and *Plasmodium* infection. Glutathione S-transferase metabolic resistance is potentially increasing the vectorial capacity of resistant *An*. *funestus* mosquitoes. This could result in a possible exacerbation of malaria transmission in areas of high GSTe2-based metabolic resistance to insecticides.

## Introduction

Malaria control in Africa mainly relies on insecticide-based interventions such as long-lasting insecticidal nets (LLINs) and indoor residual spraying (IRS)^1^. A significant decrease in the incidence of malaria was reported between 2000 and 2015, with about 70% of this success credited to insecticide-based vector control tools^1^. However, the emergence of insecticide resistance in vector populations resulting from widespread use of insecticides in public health, combined with pesticide use in agriculture, is a major problem that is jeopardizing the control of malaria^2^. The two main resistance mechanisms are target-site resistance (e.g. knockdown resistance, *kdr*) and metabolic resistance through over-expression of detoxification genes (e.g. cytochrome P450s, glutathione S-transferases and esterases)^3^. Insecticide resistance genes are often associated with pleiotropic effects on mosquito life-history traits^4,5^ which can modify their capacity to transmit parasites to different hosts^4,6^. The fitness cost of resistant alleles could affect various vector life-history traits, such as adult longevity, biting behavior, and vector competence^7^, which are important components of the vectorial capacity to transmit pathogens. However, despite the widespread distribution of resistance, its impact on malaria transmission remains unclear in many malaria vectors including *Anopheles funestus*. This is particularly true for metabolic resistance mechanisms since no molecular markers were previously available to assess such an impact, in contrast to target-site resistance (such as knockdown resistance *kdr*) for which DNA-based diagnostic tools have been available for many years^8^.

A better understanding of resistance mechanisms including metabolic resistance and, more importantly, their impact on vector life traits and disease transmission, is essential to design successful resistance management strategies^9^. A decrease in the ability of resistant mosquitoes to transmit malaria may mean insecticide resistance is not detrimentally impeding the control of this disease^7^. Conversely, if insecticide resistance increased the ability of resistant mosquitoes to infect humans, this would lead to increased malaria transmission. Only a few studies mostly, in *An*. *gambiae* s.s, have investigated the impact of resistance on vectorial capacity^4,6,10,11^. For example, the study of Kabula *et al*. (2016) in Tanzania based on the target-site resistance *kdr* marker^11^, showed that the infection of field populations of *An*. *gambiae* s.s. by *Plasmodium* parasites was significantly associated with *vgsc-1014* point mutations. Insecticide resistance was recently shown to affect the vector competence of this same mosquito species for *P*. *falciparum* field isolates as a higher prevalence of infection was observed for mutations associated with insecticide resistance^10^. The impact of resistance on vectorial capacity has yet to be examined for metabolic resistance which is the most common resistance mechanism in mosquitoes. Metabolic resistance has consistently been reported to be the main driver of pyrethroids and DDT resistance in the malaria vector *An*. *funestus*. No *kdr* mutation has been detected so far in this species^12^ despite the widespread report of insecticide resistance in *An*. *funestus* populations across Africa.

Indeed, pyrethroid resistance has been reported in various *An*. *funestus* populations including southern [Mozambique^13,14^, Malawi^15,16^], eastern [Uganda and Kenya^17,18^ and Tanzania^19^], central [Cameroon^20,21^], and western Africa [Benin^22,23^, Ghana^24,25^, Senegal^26^ and Nigeria^23^]. Noticeably, resistance to pyrethroids and DDT in these populations is consistently conferred by detoxification enzymes including glutathione S-transferases (GSTs) and cytochrome P450s. The predominance of metabolic resistance in this species makes it suitable to investigate the impact of metabolic resistance on malaria transmission. The detection of a single amino acid change (L119F) in the glutathione S-transferase epsilon 2 (GSTe2) gene conferring DDT/pyrethroid resistance in *An*. *funestus*^27^, further offers the opportunity to assess this impact. In addition, the presence of target site mutations in *An*. *funestus* such as the A296S-RDL associated with dieldrin resistance^20^ and N485I-Ace1 mutation associated with bendiocarb resistance^28^ also allows to compare the effect of metabolic resistance to that caused by target-site resistance on vectorial capacity of this vector.

Therefore, to assess the potential impact of metabolic resistance on malaria transmission, we investigated the association between the L119F-GSTe2 metabolic resistance marker and the natural infection of *Plasmodium* parasites in two pyrethroid and DDT resistant *An*. *funestus* populations from Cameroon. We established that the 119F-GSTe2 resistance allele is significantly associated with *Plasmodium* infection in resistant mosquitoes.

## Results

### Field collection and mosquito species identification

One thousand blood-fed female mosquitoes were collected in Obout and 1,147 in Mibellon after a week of collection in each site and in at least ten houses randomly selected in each village. Molecular identification of mosquitoes collected in both localities revealed that 95% of the mosquitoes belonged to the *An*. *funestus* group. The remaining 5% of mosquitoes were from *An*. *gambiae* species complex. Nearly all the *An*. *funestus* mosquitoes belonged to *An*. *funestus s*.*s* as only one mosquito belonging to another species of the *An*. *funestus* group (*An*. *leesoni)* was detected in Mibellon.

### Infection rate of *An*. *funestus* by *Plasmodium* parasites

**Figure 1:**
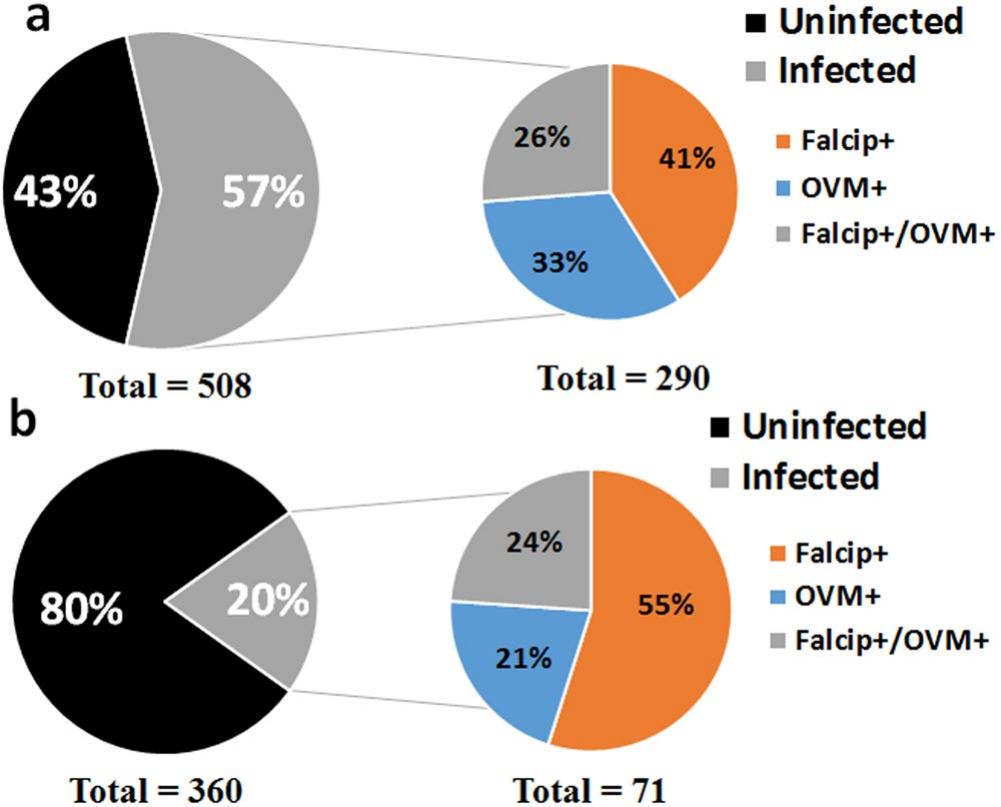
*Plasmodium* infection pattern in Obout (**a**) and Mibellon (**b**) falcip+, infection by *Plasmodium falciparum*; falcip+/OVM+, Co infection by *Plasmodium falciparum* and *P*. *ovale/vivax/malariae*; OVM+, infection by *P*. *ovale/vivax/malariae*.

In Obout, a total of 508 females (whole mosquitoes) randomly selected from the field collected individuals were tested for *Plasmodium* infection. The overall *Plasmodium* infection rate was very high in this locality with a total prevalence of 57.1% (Fig. 1a). Among the mosquitoes tested, 23% (119/508) were infected with *P*. *falciparum* (falcip+), 19% (95/508) were infected with *P*. *ovale/vivax/malariae* (OVM+), while 14.7% (76/508) were co-infected with both falcip+ and OVM+ (Table 1). In addition, the head plus thorax and abdomen were analyzed separately in 81 field-collected female mosquitoes to assess the proportion of mosquitoes harboring the infective stage of the parasite (sporozoite) and those having the oocysts. This is because sporozoites are predominantly present in the salivary glands of mosquitoes. TaqMan assay revealed a sporozoite infection rate of 14.8% (12/81) including 9.9% (8/81) falcip+, 2.5% (2/81) falcip+/OVM+ and 2.5% (2/81) OVM+. Oocysts were detected in 30.8%(25/81) mosquitoes including 19.7% (16/81) falcip+, 3.7% (3/81) falcip+/OVM+ and 7.4% (6/81) OVM+. The nested PCR performed on all the infected mosquitoes confirmed all the 16 falcip+ by Taqman (Figure S1a) whereas the three falcip+/OVM+ mosquitoes were co-infected with *P*. *falciparum* and *P*. *malariae*. Out of 18 OVM+ by Taqman, 14 were infected with *P*. *malariae* (Figure S1b) and the four remaining were not confirmed^29^. This observation indicates that in this locality, *P*. *falciparum*, and *P*. *malariae* are in circulation.

**Table 1:** Status of infection by *Plasmodium* parasites in whole mosquitoes

Localities	N	*Plasmodium* infection			
		Falcip+	Falcip+/OVM+	OVM+	Total infection
Obout	508	119 (23%) [20–27%]	76 (15%) [12–18%]	95 (19%) [15–22%]	290 (57.1%) [52.7–61.3%]
Mibellon	360	39 (11%) [8–14%]	17 (5%) [3–7%]	15 (4%) [2–7%]	71 (19.7%) [15.9–24.1%]

Abbreviations: N, total number of mosquitoes tested; Falcip+, infection by *Plasmodium falciparum*; Falcip+/OVM+, Co-infection by *P. falciparum* and *P*. *ovale/vivax/malariae*; OVM+, infection by *P*. *ovale/vivax/malariae*.

In Mibellon, out of the 360 whole mosquitoes tested (randomly selected from the total mosquitoes collected), 19.7% (71/360) were infected with *Plasmodium* parasites including 10.8% (39/360) falcip+, 4.2% (15/360) OVM+ and 4.7% (17/360) co-infection falcip+/OVM+ (Fig. 1b). Among the 60 mosquitoes dissected for head/thorax and abdomen, 20% (12/60) were oocyst-positive and 5% (3/60) sporozoite-positive with 3.3% (2/60) falcip+, 0% (0/60) falcip+/OVM+ and 1.7% (1/60) OVM+. The nested PCR validation of the TaqMan assay for oocyst positive mosquitoes confirmed all the six falcip+ whereas the two co-infected (falcip+/OVM+) were confirmed as *P*. *falciparum* and *P*. *malariae-*positive. From the four OVM-positive by Taqman assay, two were infected with *P*. *ovale and* two with *P*. *malariae* showing that *P*. *falciparum*, *P*. *ovale* (Figure S1c) and *P*. *malariae* are all present in Mibellon.

In both locations, a significant difference was found between mosquitoes harboring the oocyst stage of the parasite and those with the infective sporozoite stage (χ^2^ = 5.82; P = 0.01 in Obout and χ^2^ = 6.12; P = 0.01 in Mibellon).

### Association between L119F-GSTe2 mutation and total *Plasmodium* infection

**Figure 2:**
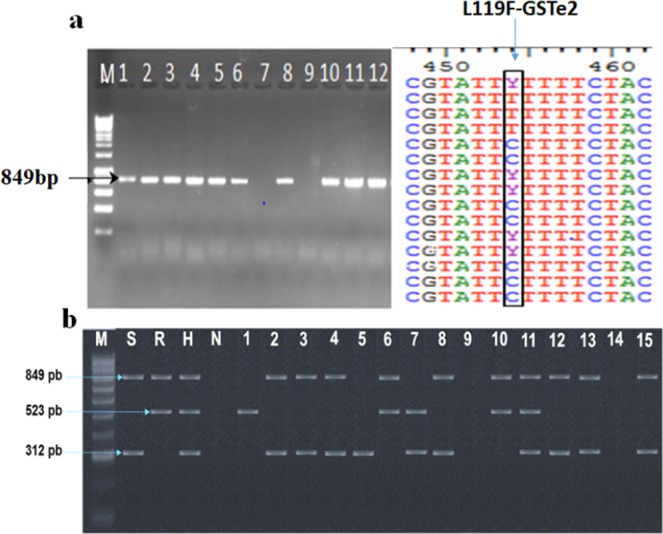
Design of a new AS-PCR for genotyping the L119F-GSTe2 mutation. (**a**) Amplification by PCR of *GSTe2* gene in *An*. *funestus* s.s. and an overview of the polymorphism of the *GSTe2* gene at the L119F point mutation where Y represents the heterozygote genotype C/T. (**b**) Agarose gel of AS-PCR to detect the L119F gste2 mutation in *An*. *funestus* s.s. Top band 849 bp, fragment common of all genotypes; the middle (523 bp) and the bottom (312 bp), resistant and susceptible mosquitoes respectively; heterozygote mosquitoes, 523 bp and the bottom 312 bp fragments. M: Molecular ladder 100 bp; positive controls (S: homozygous susceptible, R: homozygous resistant and H: heterozygote); N: negative control; 1–15: samples genotyped (1, 6, 10: homozygous resistant; 7, 11: heterozygote; 2–5, 8, 12, 13, 15: homozygous susceptible; 9, 14: no amplification).

**Figure 3:**
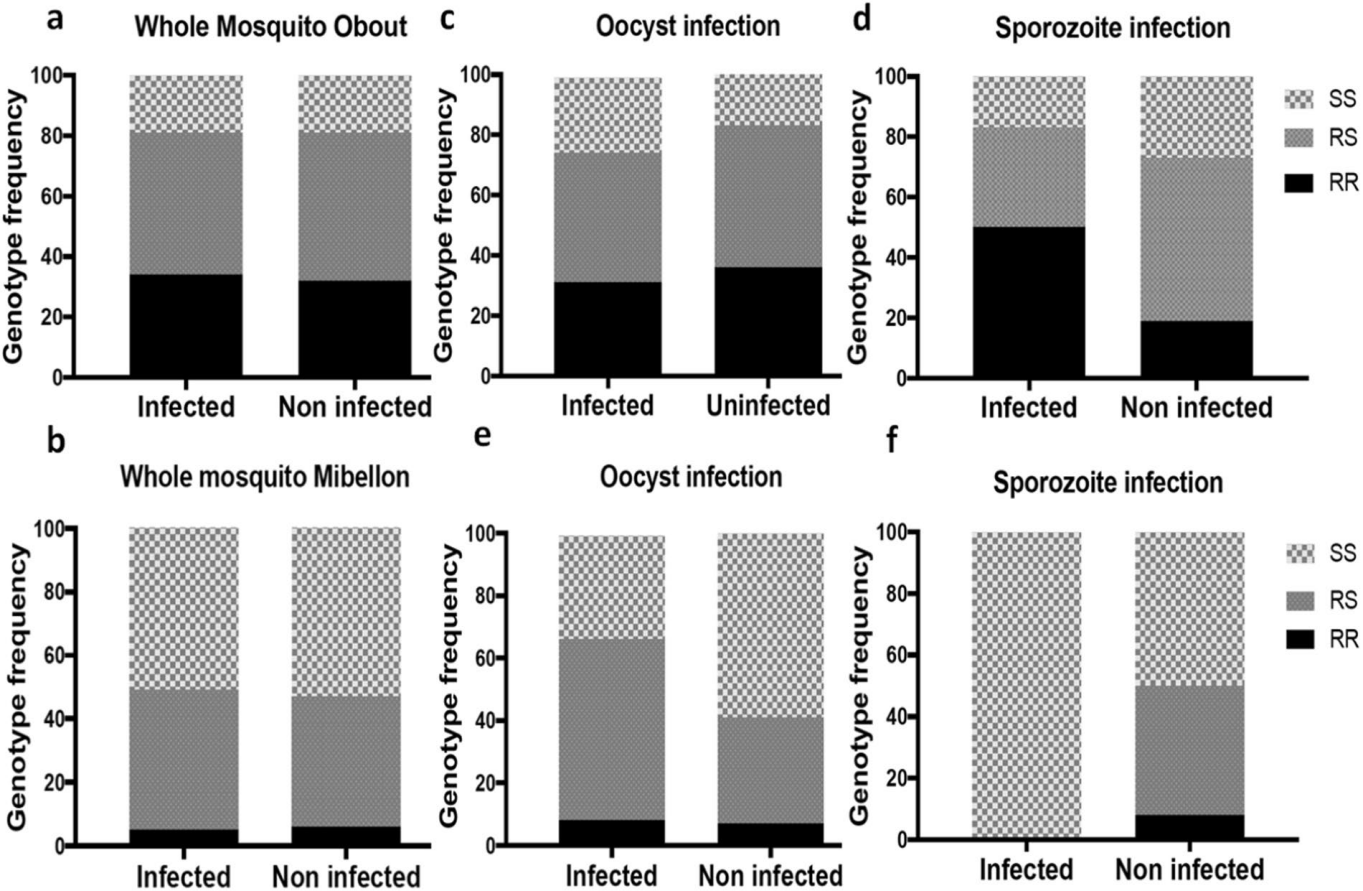
Impact of the GSTe2 glutathione S-transferase metabolic resistance (L119F-GSTe2) on the infection and transmission patterns of *Plasmodium* parasites in natural *Anopheles funestus s*.*s*. populations (**a**,**c**,**d**); are whole mosquitoes, oocyst and sporozoite infection respectively in southern Cameroon (Obout); (**b**,**e**,**f**); whole mosquitoes, oocyst and sporozoite infection respectively in Central (Mibellon).

In total, 174 whole mosquitoes (infected and uninfected randomly selected from the 508 tested above) from Obout were genotyped for the L119F-GSTe2 mutation (Table 2). All genotypes were successfully detected and later validated by direct sequencing, supporting the robustness of the new designed Allele Specific-PCR assay (Fig. 2a,b). The 119F-GSTe2 resistant allele was found at a frequency of 56.8% when combining both infected and uninfected mosquitoes. For infected mosquitoes, 33.7% were 119F/F homozygous resistant, 47.2% L119F-RS heterozygote, and 19.1% L/L119 homozygote susceptible (Fig. 3a). A similar distribution of the three genotypes was observed for uninfected mosquitoes (x^2^ = 0.34; P = 0.82) with 31.7% for 119F/F, 49.4% for L119F-RS and 18.8% for L/L119 susceptible genotype. The lack of significant correlation between L119F-GSTe2 genotypes and whole mosquito *Plasmodium* infection was further supported by odds-ratio estimates (Table 2).

**Table 2:** Distribution of L119F-GSTe2 genotypes according to *plasmodium* infection.

	phenotype	N	L119F GSTe2 genotypes			Statistic test	*p value*
			RR	RS	SS		
Obout	Infected	89	30	42	17	x^2^ = 0.34	0.82
	non infected	85	27	42	16		
	% infection		52.6%	50%	51.5%		
Mibellon	Infected	41	2	18	21	x^2^ = 0.11	0.94
	non infected	143	8	59	76		
	% infection		20%	23.3%	21.6%		

N, total number of mosquitoes successfully genotyped; RR, homozygous resistant; RS, heterozygous; SS, homozygous susceptible.

In Mibellon, 184 whole mosquitoes randomly selected out of the 360 field- collected females tested above were successfully genotyped. The overall frequency of 119F-GSTe2 resistant allele was 26.3% in both groups of mosquitoes (infected and uninfected). No difference was found in the distribution of the L119F-GSTe2 genotypes between infected and uninfected mosquitoes (*X*^2^ = 0.1 P = 0.95) (Fig. 3b) (Table 2).

### Association between L119F-GSTe2 and oocyst and sporozoite infection rates

In Obout, the distribution of L119F-GSTe2 genotypes in mosquitoes found positive for oocysts by TaqMan was as follows: 28% (7/25) homozygous resistant (119F/F), 40% (10/25) heterozygotes (L119F-RS) and 32% (8/25) homozygous susceptible (L/L119F) (Fig. 3c). No significant difference was observed in the distribution of L119F-GSTe2 genotypes between infected and uninfected mosquitoes (*X*^2^ = 3.58 P = 0.17) (Table 3). At the sporozoite stage, 50% (6/12) of infected mosquitoes were homozygous resistant, 33% (4/12) were heterozygotes and 17% (2/12) were homozygous susceptible (Fig. 3d) (Table 3). Contrary to the oocyst stage, a significant difference was observed in the distribution of L119F-GSTe2 genotypes (using the proportions) between infected and uninfected mosquitoes (X^2^ = 9.79 P = 0.007). Assessing the odds-ratio between sporozoite infected and uninfected mosquitoes showed that, homozygous resistant mosquitoes were significantly more likely to be infected with sporozoites compared to both heterozygote (OR = 2.10; IC 95%: 1.11–3.97; P = 0.013) and homozygous susceptible (OR = 2.46; IC 95%: 1.15–5.26; P = 0.012) mosquitoes. There was no difference between heterozygote and susceptible mosquitoes (OR = 1.17; P = 0.41) (Table 4).

**Table 3:** Distribution of L119F-GSTe2 genotypes between mosquitoes infected with *Plasmodium* and the prevalence of infection at both oocyst and sporozoite stage in Obout and Mibellon (Fisher Exact probability test based on the proportions).

	Phenotype	N	RR	RS	SS	Statistic test	*P value*
**Obout**							
Oocyst	Infected	**25**	7	10	8	x^2^ = 3.58	0.17
	Uninfected	**56**	19	23	14		
	**% infection**		**29**.**9%**	**30**.**3%**	**36%**		
Sporozoite	Infected	**12**	6	4	2	x^2^ = 9.79	0.007*
	non infected	**151**	20	28	17		
	**% infection**		**23**.**1%**	**12**.**5%**	**10**.**5%**		
**Mibellon**							
Oocyst	Infected	**11**	1	7	4	x^2^ = 13.05	0.001*
	non infected	**14**	3	14	24		
	**% infection**		**10%**	**38**.**1%**	**18**.**75%**		
**Sporozoite**	Infected	**3**	0	0	3	NA	NA
	non infected	**49**	4	21	25

**Table 4:** Assessment of the association of different L119F-GSTe2 genotypes with *Plasmodium* infection status in Obout and Mibellon.

Genotypes	Whole mosquitoes		Oocyst infection		Sporozoite infection	
	Odds ratio	P-value	Odds ratio	P-value	Odds ratio	P-value
**Obout**						
RR vs RS	1.11 (0.59–2.07)	0.43	0.7 (0.41–1.52)	0.30	2.10 (1.11–3.97)	**0**.01*
RR vs SS	1.06 (0.48–2.36)	0.52	0.64 (0.18–2.19)	0.34	2.46 (1.15–5.26)	**0**.01*
RS vs SS	0.96 (0.45–2.03)	0.53	0.62 (0.31–1.25)	0.12	1.17 (0.54–2.51)	0.41
**Mibellon**						
RR vs RS	0.77 (0.22–2.740)	0.47	0.67 (0.22–2.01)	0.33	NA	—
RR vs SS	0.86 (0.25–3.01)	0.54	1.92 (0.66–5.95)	0.17	NA	—
RS vs SS	1.11 (0.63–1.92)	0.41	2.96 (1.62–3.58)	0.0002*	NA	—

In Mibellon, the 20% (12/60) of mosquitoes positive for oocysts were comprised of 8.3% (1/12) 119F/F homozygous resistant, 58.3% (7/12) L119F-RS heterozygotes and 33.3% (4/12) L/L119 homozygous susceptible (Fig. 3e). A significant difference was observed in the distribution of L119F-GSTe2 genotypes between infected and uninfected mosquitoes (*X*^2^ = 13.05 P = 0.001) with L119F-RS heterozygote mosquitoes the most often infected (Table 3). Assessing the odd ratio showed no difference between the two groups (Table 4). At the sporozoite stage, only 5% (3/60) of mosquitoes were sporozoite positive and all were genotyped to be homozygous susceptible (Fig. 3f) (Table 3). No further comparisons were done because of low sample size.

### Association between A296S–RDL mutation and *Plasmodium* infection

**Figure 4:**
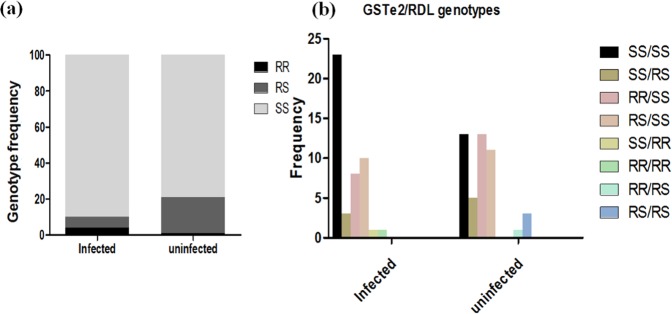
Impact of A296S-RDL target-site mutation on *Plasmodium* infection: distribution of genotype (**a**) RDL and (**b**) combinations GSTe2 /RDL)) between infected and uninfected whole mosquitoes.

The RDL mutation was genotyped using gDNA extracted from 100 and 142 whole female mosquitoes from Obout and Mibellon respectively. In Obout, all mosquitoes carried the resistant allele with a very high frequency of homozygous resistant 296 S/S genotypes (97%). In contrast, in Mibellon, only 22 (15.5%) of the mosquitoes examined had the A296S-RDL mutation including 4 (2.8%) 296S/S homozygous resistant, 18 (12.7%) A296S-RS heterozygotes and 120 homozygote susceptible (Fig. 4a). In addition, the A/A296 homozygous susceptible were present in a higher proportion among the infected mosquitoes (90%) compared to uninfected mosquitoes (79%). Assessment of the odd ratio demonstrated that mosquitoes that were A/A296 homozygous susceptible were more likely to be *Plasmodium* positive compared to other genotypes (OR = 4; IC 95%: 1.24–12.86; P = 0.03) (Table S1).

### Combined impact of L119F-GSTe2 and A296S-RDL mutations on *Plasmodium* infection

In total, 46 samples of each batch (infected and uninfected mosquitoes) were used to assess the combined impact of the two resistance mechanisms on *Plasmodium* infection in in *An*. *funestus* mosquitoes (Fig. 4b). The most prevalent combinations between both groups were (SS/SS), (RR/SS), (RS/SS) for *GSTe2*/*RDL*. The SS/RR and RR/RR combinations were present only among the infected mosquitoes whereas the RR/RS and RS/RS combinations were observed only among the uninfected mosquitoes. Nevertheless, no statistically significant differences were detected (χ^2^ = 10.5; P = 0.161). A significant difference was observed when comparing the odds ratio at the sporozoite stage between RR/RR vs SS/RR (OR = INF; P < 0.0001) and RS/RR vs SS/RR (OR = INF; P = 0.003) indicating that double homozygote mosquitoes (RR/RR) were more likely to be infected. However, no significant difference was observed at oocyst stage P ≥ 0.16) (Table S2). This supports the role of the L119F-GSTe2 allele in the ability of the mosquitoes to develop the parasite until the infective stage.

### Association between GSTe2 polymorphism and *Plasmodium* infection

#### Genetic diversity of GSTe2

**Figure 5:**
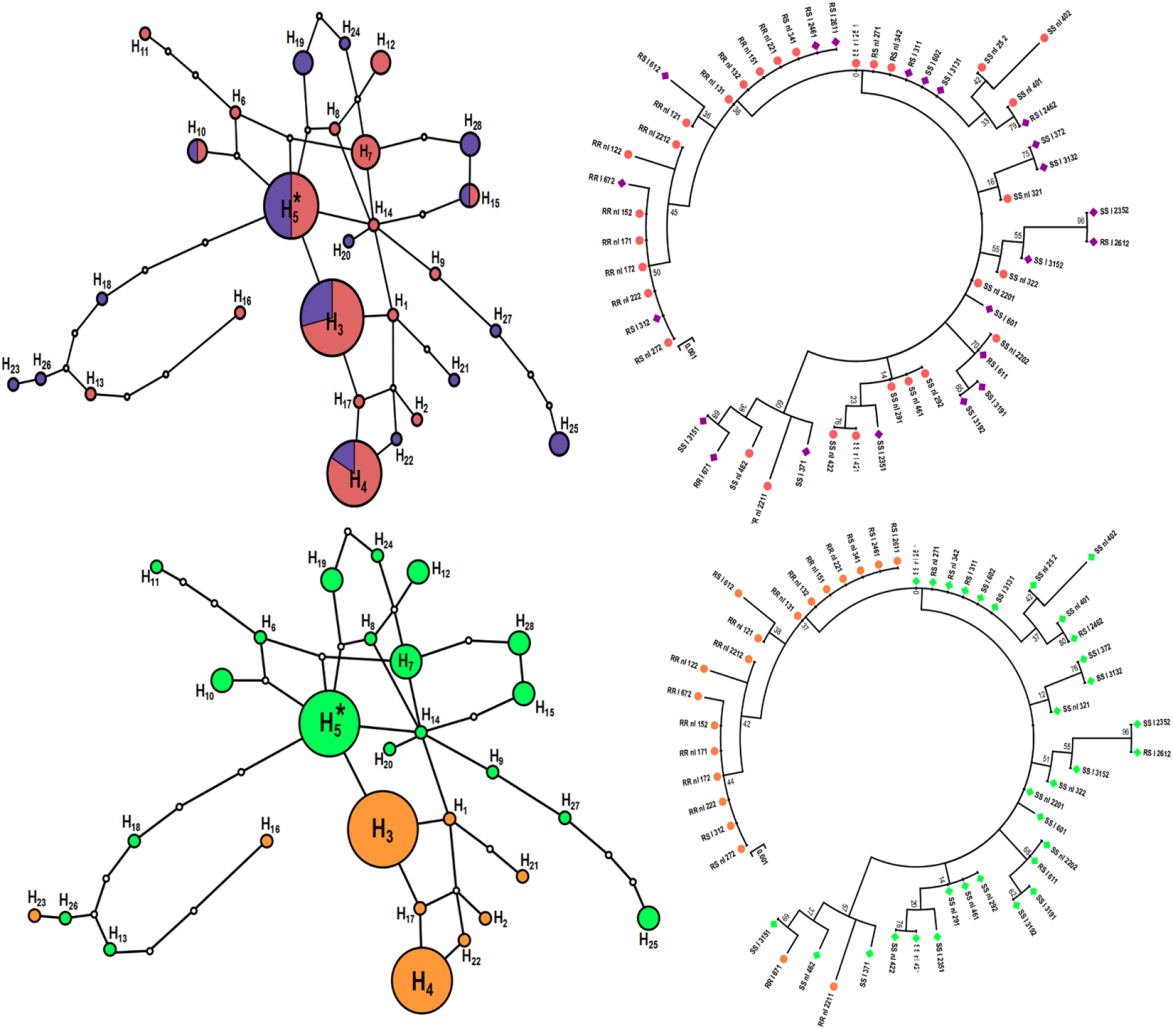
Genetic diversity parameters of *GSTe2* in *An*. *funestus* s.s. from Mibellon in relation to *Plasmodium* infection. (**a**) haplotype network and (**b**) phylogenetic tree (using a maximum likelihood method) between infected and uninfected mosquitoes; (**c**) haplotype network and (**d**) phylogenetic tree (using a maximum likelihood method) between 119F resistant allele and L119 susceptible allele.

The full length of the *GSTe2* gene (881 bp) was successfully sequenced in 26 whole mosquitoes from Mibellon including 11 infected and 15 uninfected (Fig. 5a). The genetic diversity parameters are given in Table S3, according to the status of infection and genotypes. Overall, 23 polymorphic sites defining 28 haplotypes were detected corresponding to the haplotype diversity of 0.96. Heterozygous and uninfected mosquitoes showed at lower number of polymorphic sites (3) with only 3 haplotypes (hd: 0.83). The overall nucleotide diversity was 0.005 with an average number of differences between nucleotides estimated at 4.19 showing less differences between the sequences examined. In addition, negative values were obtained for Fu and Li Tajima and F * tests in many cases.

#### Distribution of haplotypes and phylogeny

Analysis of the haplotype network of the *GSTe2* gene based on L119F genotypes of the infection status shows that there are five major haplotypes (H1, H2, H3, H4 and H5) responsible for the differentiation of haplotypes in this *An*. *funestus* field population. The ancestral haplotype (H1) as well as the haplotypes (H2, H3 and H4) were identified in mosquitoes with 119 Fresistant allele. However, haplotypes H1 and H2 were found only in uninfected mosquitoes whereas haplotypes H3 and H4 were present in both infected and uninfected mosquitoes (Fig. 5b,c). Moreover, the H5 haplotype was found to be specific for mosquitoes with the L119 susceptible allele for both infected and uninfected individuals (Fig. 5d,e). Similarly, analysis of phylogeny between the haplotypes identified did not reveal any haplotype groups associated with a specific infection status. However, there was a global clustering according to the alleles associated with the L119F mutation of the *GSTe2* gene (Table S3). This suggests that there is no association between the polymorphism of the *GSTe2* gene and infection by *Plasmodium* parasite.

## Discussion

Little information exists on the impact of metabolic resistance on the ability of mosquitoes to transmit *Plasmodium* parasites. This study is among the first to assess the association between metabolic resistance and vectorial capacity of natural populations of malaria vectors. We took advantage of the recent detection of the glutathione S-transferase L119F-GSTe2 marker in *An*. *funestus* to investigate the relationship between metabolic resistance and vectorial capacity in field collected mosquitoes. Mosquitoes used were collected from the same population at the same time for them to share a common genetic background but differ only by the presence of the resistant alleles to specifically discriminate this trait. Thus, any difference observed between the three genotypes would directly be associated with the insecticide resistance alleles.

### Role of *An*. *funestus* in malaria transmission

*An*. *funestus* s.s. was found to be the most abundant mosquito species from the indoor collection in the two study sites correlating with the indoor feeding/resting behavior of this species and supported by the presence of permanent large pools of water in both locations. *An*. *funestus* s.s. is playing a major role in malaria transmission in these areas with a very high infection rate recorded at all stages including sporozoite infection rates (14.8% in Obout and 5% in Mibellon) in field collected females’ mosquitoes. The high infection rate observed in Obout and Mibellon for *An*. *funestus* s.s. is similar to high levels of infection rates recorded previously for this species across the continent such as 20%^30^ and 50%^31^ observed in Burkina Faso, 13.6%^32^ and 18%^33^ observed in Benin and 12.5% in Ghana^25^. Although, some of the variations between these rates could be attributed to the differences in the detection methods used (TaqMan, ELISA and Nested-PCR), the consistently high levels of infection support a high vectorial capacity of *An*. *funestus* across the continent. This is of great concern for malaria control as it shows that despite ongoing control interventions, the level of malaria transmission could remain high in areas where *An*. *funestus* is the dominant vector. In this study, we noticed that *P*. *falciparum* was the predominant malaria parasite in both study sites. However, the detection of other malaria parasites, although at lower frequencies, is an indication that control and elimination efforts should not ignore other *Plasmodium* species especially *P*. *malariae*.

### Distribution of L119F-GSTe2 metabolic and A2926S target site resistance markers

It was previously demonstrated that a single amino acid change (L119F) in the over-expressed GSTe2 enzyme in *An*. *funestus s*.*s* confers resistance against DDT and cross-resistance to permethrin in West Africa^27^. The L119F mutation was detected in both localities with a higher frequency of the resistant allele in Obout. This resistance mechanism could have been selected in this population either by past DDT based IRS^34^ or by the scale up of pyrethroid-based LLINs. The presence of this resistance marker at high frequency in Obout supports previous observation in northern Cameroon (Gounougou) by Menze *et al*.^21^ suggesting that this mutation is strongly associated with DDT and permethrin cross resistance in Western and Central Africa^21,27,33^. In Mibellon, the 119F-GSTe2 mutation was found at a lower frequency for the 119F-GSTe2 resistant allele (26.3%). Two hypotheses may explain this: either the mutation was recently introduced in that population or that a recent insecticide selection pressure is favoring its presence now.

The A296S-RDL mutation in the GABA receptor gene associated with dieldrin resistance is fixed in the Obout population. In contrast, this mutation was found only at very low frequency in Mibellon. The high frequency of 296S resistant allele in *An*. *funestus* field populations from Obout is intriguing since cyclodienes are no longer used for vector control in Cameroon. It has previously been shown that dieldrin resistant mosquitoes exhibit significant fitness costs including behaviour and mating competitiveness^35,36^ that should lead to a decrease in the frequency of the resistant allele from the population overtime. Therefore, reversal of the resistance was expected in this field population in the absence of dieldrin selection pressure. The persistence of this dieldrin resistance marker in the *An*. *funestus* field population from Obout may be associated with the use of pesticides in the agricultural sector such as fipronil or lindane acting on same GABA receptor as dieldrin^37^. A population of *An*. *gambiae* fully resistant to dieldrin (100% RR) was reported in 1961 in Mbalmayo, a location of south Cameroon close to Obout^38^. This suggests also that the A296S resistant allele in *An*. *funestus* population in Obout could have become fixed before the removal of dieldrin as a vector control tool, thereby limiting the possibility of reversing dieldrin resistance.

### Impact of L119F-GSTe2 metabolic and A2926S target site resistances on *Plasmodium* infection

We did not detect any significant differences between L/L119 homozygote susceptible, L119F-RS heterozygote and 119F/F homozygote resistant genotypes and *P*. *falciparum* oocyst infection. However, mosquitoes with the A/A296 *RDL* susceptible genotype were found to be more often infected with *Plasmodium* parasites. It has previously been demonstrated that insecticide resistance mechanisms may alter the vector competence of the mosquito by affecting parasite development or susceptibility of the host to infection. This trend was not observed in this study for the A296S-RDL mutation. In a study assessing the link between insecticide resistance and vector competence, Alout and collaborators demonstrated that target site mutations (*kdr* and *ace-1R*) increased the prevalence of *P*. *falciparum* infection in pyrethroid resistant *An*. *gambiae* compared to their susceptible counterparts^10^. However, despite a higher prevalence of infection, the *kdr* resistant mosquito strain was found to harbour lower malaria parasite (oocyst) load^10^. Many factors can influence the ability of mosquitoes to be successfully infected by *Plasmodium* and harbour the parasites throughout their developmental stages until the sporozoite stage. Therefore, pleiotropic effects of insecticide resistance, immunity activation and other fitness related traits may be altered. McCarroll *et al*. reported that insecticide resistance in *Culex quinquefasciatus* mosquitoes had negative effect on the parasitic worm *Wuchereria bancrofti*, which causes human lymphatic filariasis^4,39^. Furthermore, vector immunity could also be affected by insecticide resistance^7^. A possible link between resistance and mosquito immunity was observed when a gene driving pyrethroid resistance was up-regulated in the mosquito mid-gut infected with malaria parasites^40^. *An*. *gambiae* with metabolic resistance has also been shown to have increased infection rates compared to controls^10^. However, esterase metabolism or ace-1 mutation (target site resistance) did not appear to effect the infection rates or parasitic load in *Culex pipiens* mosquitoes^6^. Oxidative stress is part of the mosquito’s immune response against *Plasmodium* but may be neutralised by overproduction of GSTs. Previously, GST resistance mechanisms were shown to protect tissues from oxidative damage in plant hoppers and increase longevity in fruit flies^41^. Therefore, neutralising oxidative stress could potentially predispose mosquitoes to higher parasite infection.

### Assessing the association between the L119F-GSTe2 mutation and the ability of mosquitoes to develop malaria parasites to the sporozoite stage

The high sporozoite infection rate of 14.8% observed in Obout was similar to recent observations in some GSTe2 related insecticide resistant populations of *An*. *funestus* in Benin^33^ and in Democratic Republic of Congo^42^. On the other hand, it was higher than the infection rates recorded in other pyrethroid resistant *An*. *funestus* populations in southern Africa such as in Malawi (4.8%)^43^ or in many African countries for other malaria vectors such as *An*. *gambiae*^11,22^. This high *Plasmodium* infection rate in *An*. *funestus* highlights the active transmission of malaria in southern Cameroon by this species. Furthermore, a significant association was found between the 119F/F-GSTe2 resistant genotype and the presence of *P*. *falciparum* sporozoite in *An*. *funestus* in Obout. The sporozoite infection rate in 119F/F homozygous resistant mosquitoes was three times higher than that of the homozygous susceptible mosquitoes. This suggests that parasites developed better in resistant mosquitoes than in susceptible counterparts which should be a cause for concern as possession of this resistance allele may potentially be allowing higher malaria transmission. A similar result was previously found in *An*. *gambiae* s.s. for the target-site *vgsc*-L1014S mutation^11^. The association between *Plasmodium* sporozoite infection and GSTe2-based resistant mosquitoes observed in this study could be due to three main possibilities. First, this could be caused by the phenotypic expression of L119F-GSTe2 such that the 119F/F homozygous resistant mosquitoes could live longer due to their ability to withstand exposure to insecticides in the field. In this case they are more likely to allow the *Plasmodium* parasites to complete their extrinsic incubation period compared to homozygous susceptible mosquitoes. This suggestion is supported by the fact that glutathione S-transferases have been shown to be associated with resistance^27^ and also to protect insect tissues from the damaging effects of oxidative stress and extent life span of insects by increasing solubility and excretion of free radicals^7,44–46^. The second possibility of the higher likelihood of sporozoite infection in mosquitoes with GSTe2–119F/F genotype is that insecticide resistance could alter mosquito immunity. Indeed, it is possible as suggested previously^7^ that the over-expression of GSTs in homozygous mosquitoes could be protecting *Plasmodium* parasites against the damaging effects of reactive oxidative species (ROS). These ROS are known as key component of the mosquito immune responses against *Plasmodium* infection^47^. It is likely that over-expression of GSTs may affect parasite development or susceptibility of the mosquito to infection by neutralizing the oxidative response of the 119F/F mosquitoes to *Plasmodium* and thus potentially increasing their susceptibility to infection. This will need to be fully established possibly through experimental infection studies. Thirdly, the higher *Plasmodium* infection rate seen in homozygous resistant 119F/F mosquitoes could be due the potential reduction of immune-competence through a resource trade-off between increased over-expression of GSTs and the mosquito’s immune response. It has been shown that when certain energy resources are redirected towards the production of large amounts of detoxification enzymes such as GSTs, a resource-based trade-off is usually involved and affects the vector immuno-competence^48^. As a result, there is likely to be a depletion of energy resources which limits the vector’s ability to mount a sufficient immune response against *Plasmodium* leading to increased infection in those resistant mosquitoes as observed in our study. However, more studies are needed to establish the extent to which insecticide resistance affects the mosquito’s vectorial capacity to confirm the impact of resistance on malaria transmission.

## Conclusion

This study investigated the association between a molecular marker of GST-mediated metabolic resistance and *Plasmodium* infection in natural populations of a major malaria vector, *An*. *funestus*. The study revealed that mosquitoes that were homozygous for the resistance allele were more likely to harbor *Plasmodium* sporozoites. This suggests that the proliferation of this metabolic resistance marker could exacerbate malaria transmission in the field and thus have important public health consequences.

## Methods

### Study site and sample collection

Mosquito collections were performed in Cameroon in May 2016 and February 2017 in Obout (Southern Region, 3°28′17.0″N 11°44′09.4″E) and Mibellon (Adamaoua Region, 6°46′N, 11°70′E) for one week per site. Prior to mosquito collection, verbal consent was obtained from the village council chairpersons and from each household representative. Indoor resting female mosquitoes were collected using electric aspirators in both locations and transported to the insectary of LSTM Research Unit at OCEAC in Yaoundé, Cameroon.

### DNA extraction

Genomic DNA (gDNA) was extracted via the LIVAK method^49^. Following extraction, NanoDrop™ spectrophotometer (Thermo Scientific, Wilmington, USA) was used to determine the concentration and purity of the extracted gDNA before storage at −20 °C.

### Species identification

The females used for oviposition were morphologically identified using the key of Gillies and De Meillon^50^. Molecular identification was achieved through a cocktail polymerase chain (PCR) reaction described by Koekemoer^51^ to determine species composition of *An*. *funestus* group in the two study sites.

### Detection of *Plasmodium* parasites

A TaqMan assay described by Bass *et al*.^29^ was used to establish the *Plasmodium* infection status of field collected mosquitoes. Two probes were used in this assay. The first, labelled with FAM, detects *P*. *falciparum*, and the second, labelled with VIC, to detect *P*. *vivax*, *P*. *ovale* and/or *P*. *malariae* (OVM). Firstly, gDNA was extracted from the whole mosquitoes to assess the overall proportion of *An*. *funestus* infected by *Plasmodium* parasites in the field. Secondly, another sets of field collected mosquitoes were dissected in two parts: the abdomens, used for the detection of *Plasmodium* infection at the oocyst stage, and the head plus thorax for the assessment of sporozoite infection rate. Results of TaqMan assay were confirmed by performing a nested PCR assay as previously described^52^.

### Genotyping of the L119F-GSTe2 mutation

The L119F-GSTe2 mutation previously shown to play a major role in DDT and permethrin resistance in *An*. *funestus*^27^ was genotyped in F_0_ field-collected mosquitoes using a newly designed allele-specific PCR (AS-PCR) diagnostic assay. Two pairs of primers were needed for the AS-PCR (two outer and two inner primers). Specific primers were designed manually to match the mutation and an additional mismatched nucleotide was added in the 3^th^ nucleotide from the 3′ end of each inner primer to enhance the specificity. More details on the primer sequences are given in Table S4. PCR was carried out using 10 mM of each primer and 1ul of genomic DNA as template in 15 μl reactions containing 10X Kapa Taq buffer A, 0.2 mM dNTPs, 1.5 mM MgCl_2_, 1U Kapa Taq (Kapa biosystems). The cycle parameters were: 1 cycle at 95 °C for 2 min; 30 cycles of 94 °C for 30 s, 58 °C for 30 s, 72 °C for 1 min and then a final extension step at 72 °C for 10 min. PCR products were separated on 2% agarose gel by electrophoresis. This method detects homozygote resistant (119F/F) at 523 bp, homozygote susceptible (L119F-RS) at 312 bp, and heterozygote (L/L119) with both bands. Association between the *GSTe2* mutation and malaria transmission potential was assessed by calculating the odds ratio of sporozoite infection rate between the homozygous resistant (119F/F), heterozygote (L119F-RS) and homozygous susceptible (L/L119) individuals compared to uninfected individuals, with statistical significance was computed based on the Fisher’s exact probability test.

### Association between the genetic diversity of the *GSTe2* gene and *Plasmodium* infection in *An*. *funestus*

The entire *GSTe2* gene of 881 bp in *An*. *funestus* was amplified in 26 whole mosquitoes [11 infected by *Plasmodium* parasites (both stages) and 15 non-infected]. Two primers; GSTe2F, 5′GGA ATT CCA TAT GAC CAA GCT AGT TCT GTA CAC GCT 3′ and GSTe2R, 5′ TCT AGA TCA AGC TTT AGC ATT TTC CTC CTT 3′ were used to amplify the gene in 15 μl reaction containing 10 mM of each primer, 10X Kapa Taq buffer A, 0.2 mM dNTPs, 1.5 mM MgCl_2_, 1U Kapa Taq (Kapa biosystems). PCR conditions were 1 cycle at 95 °C for 5 min; 30 cycles of 94 °C for 30 s, 55 °C for 30 s, 72 °C for 1 min and then final extension at 72 °C for 10 min. PCR product was firstly visualized on 1.5% agarose gel stained with Midori green dye (Nippon Genetics Europe, Germany) and then purified using ExoSAP (Thermo Fisher Scientific, Waltham, MA, USA) according to manufacturer recommendations and directly sequenced on both strands. Sequences were visualized and corrected using BioEdit v7.2.5 software^53^. Alignment of these sequences was done using ClustalW Multiple Alignment integrated in BioEdit^54^. Genetic diversity parameters were assessed using DnaSP v5.10.01^55^ and MEGA v7.0.21^56^ softwares.

### Genotyping of the A296S-RDL GABA receptor mutation

To compare the role of metabolic resistance to that of target-site resistance mechanism, we genotyped the A296S-RDL mutation associated with dieldrin resistance^20^ in *Plasmodium* infected and uninfected mosquitoes. The A296S-RDL mutation was genotyped using a protocol previously described by Riveron *et al*.^43^. Furthermore, the combined effect of harboring both alleles of A296S-RDL and L119F-GSTe2 on the infection status of field collected mosquitoes was also assessed.

## Supplementary information

Supplementary Information

## References

[1] Bhatt S (2015). The effect of malaria control on Plasmodium falciparum in Africa between 2000 and 2015. Nature.

[2] Ranson H, Lissenden N (2016). Insecticide Resistance in African Anopheles Mosquitoes: A Worsening Situation that Needs Urgent Action to Maintain Malaria Control. Trends Parasitol.

[3] Alout H, Labbé P, Chandre F, Cohuet A (2017). Malaria vector control still matters despite insecticide resistance. Trends in parasitology.

[4] McCarroll L, Hemingway J (2002). Can insecticide resistance status affect parasite transmission in mosquitoes?. Journal Insect Biochemistry and Molecular Biology.

[5] Raymond, M., Berticat, C., Weill, M., Pasteur, N. & Chevillon, C. In *Microevolution Rate*, *Pattern*, *Process* 287–296 (Springer, 2001). 11838771

[6] Vezilier, J., Nicot, A., Gandon, S. & Rivero, A. Insecticide resistance and malaria transmission: infection rate and oocyst burden in *Culex pipiens* mosquitoes infected with *Plasmodium relictum*. *Malaria Journal* **9** (2010). 10.1186/1475-2875-9-379 PMC3313086 21194433

[7] Rivero A, Vézilier J, Weill M, Read AF, Gandon S (2010). Insecticide Control of Vector-Borne Diseases: When Is Insecticide Resistance a Problem?. PLoS Pathog.

[8] Martinez-Torres D (1998). Molecular characterization of pyrethroid knockdown resistance (kdr) in the major malaria vector Anopheles gambiae s.s. Insect Mol Biol.

[9] WHO (2012). Global plan for insecticide resistance management in malaria vectors (GPIRM). 2012. ISBN.

[10] Alout H (2013). Insecticide resistance alleles affect vector competence of *Anopheles gambiae* s.s. for *Plasmodium falciparum* field isolates. PLoS ONE.

[11] Kabula B (2016). A significant association between deltamethrin resistance, Plasmodium falciparum infection and the Vgsc-1014S resistance mutation in Anopheles gambiae highlights the epidemiological importance of resistance markers. Malar J.

[12] Irving H, Wondji CS (2017). Investigating knockdown resistance (kdr) mechanism against pyrethroids/DDT in the malaria vector Anopheles funestus across Africa. BMC Genet.

[13] Casimiro SL, Hemingway J, Sharp BL, Coleman M (2007). Monitoring the operational impact of insecticide usage for malaria control on Anopheles funestus from Mozambique. Malar J.

[14] Cuamba N, Morgan JC, Irving H, Steven A, Wondji CS (2010). High level of pyrethroid resistance in an Anopheles funestus population of the Chokwe District in Mozambique. PLoS One.

[15] Hunt RH, Edwardes M, Coetzee M (2010). Pyrethroid resistance in southern African Anopheles funestus extends to Likoma Island in Lake Malawi. Parasites & Vectors.

[16] Wondji CS (2012). Impact of pyrethroid resistance on operational malaria control in Malawi. Proc Natl Acad Sci USA.

[17] Morgan JC, Irving H, Okedi LM, Steven A, Wondji CS (2010). Pyrethroid resistance in an Anopheles funestus population from Uganda. PLoS One.

[18] Mulamba C (2014). Widespread pyrethroid and DDT resistance in the major malaria vector Anopheles funestus in East Africa is driven by metabolic resistance mechanisms. PLoS One.

[19] Lwetoijera DW (2014). Increasing role of Anopheles funestus and Anopheles arabiensis in malaria transmission in the Kilombero Valley, Tanzania. Malar J.

[20] Wondji CS (2011). Identification and distribution of a GABA receptor mutation conferring dieldrin resistance in the malaria vector Anopheles funestus in Africa. Insect Biochem Mol Biol.

[21] Menze BD (2016). Multiple Insecticide Resistance in the Malaria Vector Anopheles funestus from Northern Cameroon Is Mediated by Metabolic Resistance Alongside Potential Target Site Insensitivity Mutations. PLoS One.

[22] Djouaka R, Irving H, Tukur Z, Wondji CS (2011). Exploring Mechanisms of Multiple Insecticide Resistance in a Population of the Malaria Vector Anopheles funestus in Benin. PLoS One.

[23] Djouaka RJ (2016). Evidence of a multiple insecticide resistance in the malaria vector Anopheles funestus in South West Nigeria. Malar J.

[24] Okoye PN, Brooke BD, Koekemoer LL, Hunt RH, Coetzee M (2008). Characterisation of DDT, pyrethroid and carbamate resistance in Anopheles funestus from Obuasi, Ghana. Trans R Soc Trop Med Hyg.

[25] Riveron JM (2016). Multiple insecticide resistance in the major malaria vector Anopheles funestus in southern Ghana: implications for malaria control. Parasit Vectors.

[26] Samb B (2016). Investigating molecular basis of lambda-cyhalothrin resistance in an Anopheles funestus population from Senegal. Parasit Vectors.

[27] Riveron JM (2014). A single mutation in the GSTe2 gene allows tracking of metabolically-based insecticide resistance in a major malaria vector. Genome Biol.

[28] Ibrahim SS, Ndula M, Riveron JM, Irving H, Wondji CS (2016). The P450 CYP6Z1 confers carbamate/pyrethroid cross-resistance in a major African malaria vector beside a novel carbamate-insensitive N485I acetylcholinesterase-1 mutation. Mol Ecol.

[29] Bass C (2008). PCR-based detection of Plasmodium in Anopheles mosquitoes: a comparison of a new high-throughput assay with existing methods. Malar.J..

[30] Dabire KR (2007). Anopheles funestus (Diptera: Culicidae) in a humid savannah area of western Burkina Faso: bionomics, insecticide resistance status, and role in malaria transmission. J Med Entomol.

[31] Costantini C, Sagnon N, Ilboudo-Sanogo E, Coluzzi M, Boccolini D (1999). Chromosomal and bionomic heterogeneities suggest incipient speciation in Anopheles funestus from Burkina Faso. Parassitologia.

[32] Sandeu MM (2012). Optimized Pan-species and speciation duplex real-time PCR assays for Plasmodium parasites detection in malaria vectors. PLoS One.

[33] Djouaka R (2016). Multiple insecticide resistance in an infected population of the malaria vector Anopheles funestus in Benin. Parasit Vectors.

[34] Antonio-Nkondjio C (2017). Review of the evolution of insecticide resistance in main malaria vectors in Cameroon from 1990 to 2017. Parasites & Vectors.

[35] Rowland M (1991). Behaviour and fitness of γHCH/dieldrin resistant and susceptible female Anopheles gambiae and An.stephensi mosquitoes in the absence of insecticide. Medical and Veterinary Entomology.

[36] Rowland M (1991). Activity and mating competitiveness of gamma HCH/dieldrin resistant and susceptible male and virgin female Anopheles gambiae and An.stephensi mosquitoes, with assessment of an insecticide-rotation strategy. Med Vet Entomol.

[37] Tantely ML (2010). Insecticide resistance in Culex pipiens quinquefasciatus and Aedes albopictus mosquitoes from La Reunion Island. Insect Biochem Mol Biol.

[38] Gariou J, Mouchet J (1961). [Appearance of a dieldrin-resistant strain of Anopheles gambiae in the antimalaria campaign zone in southern Cameroon’s]. Bull Soc Pathol Exot Filiales.

[39] McCarroll L (2000). Insecticides and mosquito-borne disease. Nature.

[40] Felix RC, Muller P, Ribeiro V, Ranson H, Silveira H (2010). Plasmodium infection alters Anopheles gambiae detoxification gene expression. BMC Genomics.

[41] McElwee JJ (2007). Evolutionary conservation of regulated longevity assurance mechanisms. Genome Biol.

[42] Riveron JM, Watsenga F, Irving H, Irish SR, Wondji CS (2018). High Plasmodium Infection Rate and Reduced Bed Net Efficacy in Multiple Insecticide-Resistant Malaria Vectors in Kinshasa, Democratic Republic of Congo. The Journal of infectious diseases.

[43] Riveron JM (2015). Rise of multiple insecticide resistance in Anopheles funestus in Malawi: a major concern for malaria vector control. Malar J.

[44] Parkes TL, Hilliker AJ, Phillips JP (1993). Genetic and biochemical analysis of glutathione-S-transferase in the oxygen defense system of Drosophila melanogaster. Genome.

[45] Hayes JD, McLellan LI (1999). Glutathione and glutathione-dependent enzymes represent a co-ordinately regulated defence against oxidative stress. Free Radic Res.

[46] Vontas JG, Small GJ, Hemingway J (2001). Glutathione S-transferases as antioxidant defence agents confer pyrethroid resistance in Nilaparvata lugens. Biochem J.

[47] Kumar, S. *et al*. *The role of reactive oxygen species on Plasmodium melanotic encapsulation in Anopheles gambiae*. Vol. 100 (2003). 10.1073/pnas.2036262100 PMC283559 14623973

[48] Després L (2014). Chemical and biological insecticides select distinct gene expression patterns in Aedes aegypti mosquito. Biology Letters.

[49] Livak KJ (1984). Organization and mapping of a sequence on the Drosophila melanogaster X and Y chromosomes that is transcribed during spermatogenesis. Genetics.

[50] Gillies M, De Meillon B (1968). The Anophelinae of Africa South of the Sahara (Ethiopian Zoogeographical Region). Inst Med Res.

[51] Koekemoer LL, Kamau L, Hunt RH, Coetzee M (2002). A cocktail polymerase chain reaction assay to identify members of the Anopheles funestus (Diptera: Culicidae) group. Am J Trop Med Hyg.

[52] Snounou G (1993). High sensitivity of detection of human malaria parasites by the use of nested polymerase chain reaction. Molecular and Biochemical Parasitology.

[53] Hall T (1999). BioEdit: a user-friendly biological sequence alignment editor and analysis program for Windows 95/98/NT. Nucleic Acids Symposium Series.

[54] Thompson J, Higgins D, Gibson T (1994). CLUSTALW: improving the sensitivity of progressive miltiple sequence alignment through sequence weighting, position specific gap penalties and weght matrix choice. Nucleic Acids Research.

[55] Librado P, Rozas J (2009). DnaSP v5: a software for comprehensive analysis of DNA polymorphism data. Bioinformatics.

[56] Kumar S, Stecher G, Tamura K (2016). MEGA7: Molecular Evolutionary Genetics Analysis Version 7.0 for Bigger Datasets. Molecular Biology and Evolution.

