# Supplementary material for: A marker of glutathione S-transferase-mediated resistance to insecticides is associated with higher Plasmodium infection in the African malaria vector Anopheles funestus
Source: Sci Rep. 2019 Apr 8;9:5772. doi: 10.1038/s41598-019-42015-1 (PMC6453935; doi:10.1038/s41598-019-42015-1)
Supplement: Supplementary file 1 — Supplementary Information [file 41598_2019_42015_MOESM1_ESM.pdf]

A glutathione S-transferase metabolic resistance to insecticides is associated with higher *Plasmodium* infection in the African malaria vector *Anopheles funestus*

Authors and Affiliations

Magellan Tchouakui[1,2,3], Mu-Chun Chiang[4], Cyrille Ndo[1,2,5], Carine Kegoum Kuicheu[1,2,3], Nathalie Amvongo-Adjia[1,3], Murielle J. Wondji[1,2,4], Micareme Tchoupo[1,2], Jacob Miranda Riveron [1,2,4], Charles S. Wondji[1,2,4*]

[1]Research Unit LSTM/OCEAC P.O. BOX 288, Yaoundé, Cameroon

[2]Centre for Research in Infectious Diseases (CRID) P.O. BOX 13591, Yaoundé, Cameroon

[3]Department of Animal Biology and Physiology, Faculty of Science, University of Yaoundé 1, P.O. Box 812, Yaoundé, Cameroon

[4]Department of Vector Biology, Liverpool School of Tropical Medicine, Pembroke Place, L35QA, Liverpool, UK

[5]University of Douala, P.O. Box 2701, Douala, Cameroon

[*]corresponding author; email address: charles.wondji@lstmed.ac.uk

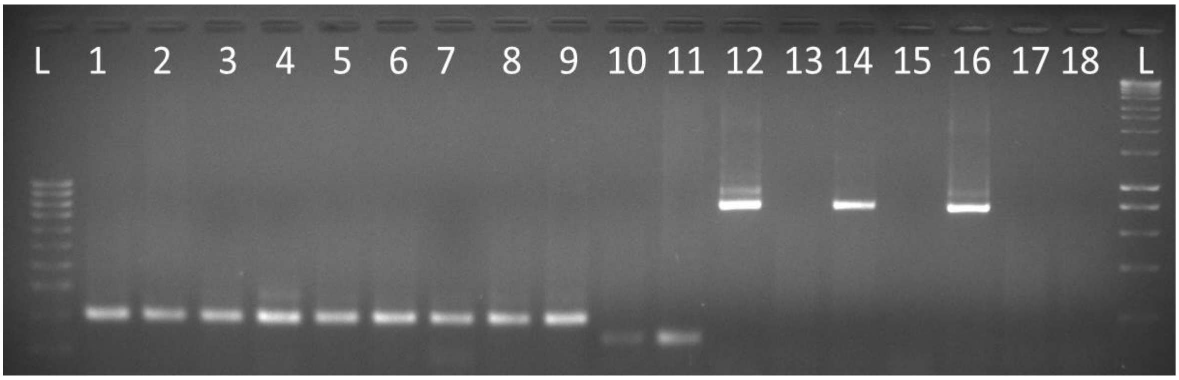

Figure S1 : Nested PCR on mosquitoes positive by Taqman;   L= ladder 100bp-1kb, 1-9 = *P. falciparum* (205bp)*;* 10-11 = *P. malariae* (144bp)*;* 12, 14, 16 = *P. ovale* (800bp), 13, 15, 17 =No amplification; 18= negative control; L= ladder 0.1-10kb. This gel image was cropped from the original gel.

```
HAP      Freq   00111111123333344444445   Infection
                77112345938899900333353    status
                06594918084501456046746
H1(R)    1      TTGAACAAGGTTACGAGTCCATA     nI
H2(R)    1      ...T........G..........     nI
H3(R)    7      ...............G.......     nI,I
H4(R)    6      G..T...........G.......     nI,I
H5(S)    6      ...............G.....C.     nI,I
H6(S)    1      ..C............G.....CC     nI
H7(S)    3      .....................CC     nI
H8(S)    1      .......G.............C.     nI
H9(S)    1      ......G..............C.     nI
H10(S)   2      ..C...........AG.....C.     nI,I
H11(S)   1      ..C....G....G..G.C...CC     nI
H12(S)   2      .......G.T...........CC     nI
H13(S)   1      .......G..........TTTCC     nI
H14(S)   1      .....................C.     nI
H15(S)   2      .....T.......A.......C.     nI,I
H16(R)   1      .......G.T........TTT..     nI
H17(R)   1      ...T...........G.......     nI
H18(S)   1      ...............G..TTTC.     I
H19(S)   2      .......GA......G.....C.     I
H20(S)   1      ................C....C.     I
H21(R)   1      .A..C..................     I
H22(R)   1      .....T............TTT.C     I
H23(R)   1      G..T...................     I
H24(S)   1      .......GA............CC     I
H25(S)   2      G.C...G...AC.........C.     I
H26(S)   1      .....T............TTTCC     I
H27(S)   1      ..C...G..............C.     I
H28(S)   2      .....T.......A.......CC     I
```

Figure S2: Polymorphic sites of the GSTe2 gene sequences between infected and non-infected *An. funestus* mosquitoes in Mibellon. nI is for non-infected; I is for infected. The number in red indicate the nucleotide position of the substitution site. Each line represents an haplotype (H) whereas R or S stand for resistant or susceptible haplotype.

Table S1: Assessment of association between different genotypes for RDL mutation on infection by *Plasmodium spp*. in *Anopheles funestus* s.s in Mibellon as RDL is nearly fixed in Obout for whole mosquitoes.

**Génotypes**	***Rdl***	
	**Odds ratio**	**P-value**
**RR vs RS**	0,10<br>(0,10 – 1,19)	0,08
**RR vs SS**	0,38<br>(0,04 – 3,77)	0,37
**RS vs SS**	4<br>(1,24 – 12,86)	0,03*

Abbreviations: RR, homozygous resistant; RS, heterozygous; SS, homozygous susceptible; for odds ratio, confidence interval at 95% are given in brackets; *,significant.

Table S2: Assessment of association between different genotypes for RDL combined with GSTe2 mutations on infection by *Plasmodium spp*. in *Anopheles funestus* s.s.

Genotypes	***Oocyst infection***		***Sporozoite infection***	
	**Odds ratio**	**P-value**	**Odds ratio**	**P-value**
**RR/RR vs RS/RR**	1.69 (0.7 – 2.19)	0.318	2.42 (0.76 – 2.8)	0.29
**RR/RR vs SS/RR**	2.7 (0.8-3.9)	0.16	Inf (inf)	<0.0001
**RS/RR vs SS/RR**	1.59 (0.58 – 3.1)	0.36	Inf (inf)	0.003

Table S3: genetic diversity parameters of *GSTe2* sequences according to the infection status and the L119F genotypes

Samples	N	S	h (Hd)	Syn	NSyn	π (k)	D	F*
P. falciparum								
Infected	22	20	16 (0,97)	4	7	0,006 (4,90)	- 0,39^{ns}	0,26^{ns}
uninfected	30	17	17 (0,94)	4	5	0,005 (3,64)	- 0,52^{ns}	- 0,35^{ns}
GSTe2								
Resistant (RR)	14	11	8 (0,87)	3	3	0,004 (3,03)	- 0,49^{ns}	- 0,56^{ns}
Heterozygote (RS)	12	13	7 (0,89)	3	5	0,004 (3,30)	- 0,99^{ns}	- 1,17^{ns}
Susceptible (SS)	26	19	19 (0,97)	5	3	0,005 (3,98)	- 0,71^{ns}	- 0,71^{ns}
GSTe2 × *P. falciparum*								
RR × *P. fal*+	2	7	2 (1)	2	2	0,009 (7)	//	//
RR × *P. fal*-	12	9	6 (0,82)	2	2	0,003 (2,44)	- 0,74^{ns}	- 1,22^{ns}
RS × *P. fal*+	8	13	7 (0,96)	3	5	0,005 (4,21)	- 0,81^{ns}	- 0,96^{ns}
RS × *P. fal*-	4	3	3 (0,83)	1	2	0,002 (1,67)	0,17^{ns}	0,15^{ns}
SS × *P. fal*+	12	15	9 (0,96)	4	3	0,006 (4,73)	- 0,21^{ns}	0,27^{ns}
SS × *P. fal*-	14	14	11 (0,96)	3	3	0,004 (3,37)	- 0,95^{ns}	- 1,41^{ns}
Total	52	23	28 (0,96)	5	7	0,005 (4,19)	- 0,57^{ns}	0,07^{ns}

N : number of sequences analysed ; (2n) ; S : number of polymorphic sites; h : number of haplotypes (dh : haplotypic diversity) ; Syn : number of synonymous mutations ; Nsyn : number of non-synonymous mutations; π: nucléotidique diversity (k : mean number of nucléotidic differences between sequences) ; D: Tajima index ; F* : Fu & Li index. ; ns : no significant

Table S4: Details of primer sequences used to analyse for *L119F GSTe2* mutation.

Primers	Sequence (5' to 3')
Ndel_Gste2F	GGAATTCCATATGACCAAGCTAGTTCTGTACACGCT
Xbal_Gste2 R	TCTACATCAAGCTTTAGCATTTTCCTCCTT
L119F-Res	CGGGAATGTCCGATTTTCCGTAGAA**t**A**A**
L119-F-Sus	CATTTCTTATTCTCATTTACAGGAGCGTA**a**T**C**
